# Supplementary material for: Making sense of and working with COVID-19 related guidelines and information in Danish general practice–A qualitative study
Source: PLoS One. 2023 Feb 10;18(2):e0281579. doi: 10.1371/journal.pone.0281579 (PMC9916589; doi:10.1371/journal.pone.0281579)
Supplement: Attachment — Submitted filename: Response to Reviewers.docx [file pone.0281579.s001.docx]

**Response to reviewers**

Our point by point responses are described in the table below.

Thank you.

| **Comment** | **Response** |
| --- | --- |
| *Journal requirements:* |  |
| When submitting your revision, we need you to address these additional requirements.  1. Please ensure that your manuscript meets PLOS ONE's style requirements, including those for file naming. The PLOS ONE style templates can be found at  https://journals.plos.org/plosone/s/file?id=wjVg/PLOSOne_formatting_sample_main_body.pdf and  https://journals.plos.org/plosone/s/file?id=ba62/PLOSOne_formatting_sample_title_authors_affiliations.pdf | The guidelines have been read and changes have been made to comply with the requirements regarding headlines. |
| 2. "When reporting the results of qualitative research, we suggest consulting the COREQ guidelines: <http://intqhc.oxfordjournals.org/content/19/6/349>.  In this case, please consider including more information on the number of interviewers, their training and characteristics. | As described in line 112-113 JHA and TDD conducted the interviews. We have added information about our characteristics in line 102-105 |
| 3. We note that the grant information you provided in the ‘Funding Information’ and ‘Financial Disclosure’ sections do not match.  When you resubmit, please ensure that you provide the correct grant numbers for the awards you received for your study in the ‘Funding Information’ section.  4. Thank you for stating the following in the Acknowledgments Section of your manuscript:  “We would like to thank all the participating GPs for their time and contributions.”  We note that you have provided funding information that is not currently declared in your Funding Statement. However, funding information should not appear in the Acknowledgments section or other areas of your manuscript. We will only publish funding information present in the Funding Statement section of the online submission form.  Please remove any funding-related text from the manuscript and let us know how you would like to update your Funding Statement. Currently, your Funding Statement reads as follows:  “The author(s) received no specific funding for this work. The participating GPs were remunerated for the interviews with a grant from The Committee of Multipractice Studies in General Practice”  Please include your amended statements within your cover letter; we will change the online submission form on your behalf. | Both ‘Funding Information’ and ‘Financial Disclosure’ sections should state: “The authors received no specific funding for this work.”  In the manuscript we submitted we cannot identify any information about funding in the Acknowledgments section. We have deleted information about remuneration in the method section.  We received a grant solely for remuneration of the participating GPs. We are unsure where this should be stated and hope you can change this for us.  It should state: “The participating GPs were remunerated for the interviews with a grant from The Committee of Multipractice Studies in General Practice (Grant number: EMN: 2020.01302-1388655)”  This information is also provided in the cover letter |
| 5. We note that you have indicated that data from this study are available upon request. PLOS only allows data to be available upon request if there are legal or ethical restrictions on sharing data publicly. For more information on unacceptable data access restrictions, please see http://journals.plos.org/plosone/s/data-availability#loc-unacceptable-data-access-restrictions.  In your revised cover letter, please address the following prompts:  a) If there are ethical or legal restrictions on sharing a de-identified data set, please explain them in detail (e.g., data contain potentially sensitive information, data are owned by a third-party organization, etc.) and who has imposed them (e.g., an ethics committee). Please also provide contact information for a data access committee, ethics committee, or other institutional body to which data requests may be sent.  b) If there are no restrictions, please upload the minimal anonymized data set necessary to replicate your study findings as either Supporting Information files or to a stable, public repository and provide us with the relevant URLs, DOIs, or accession numbers. For a list of acceptable repositories, please see http://journals.plos.org/plosone/s/data-availability#loc-recommended-repositories.  We will update your Data Availability statement on your behalf to reflect the information you provide. | We have reread the PLOS ONE Data availability policy and believe we with this qualitative study comply with the requirements. According to Danish GDPR policies we cannot make more data available. The interviewees where promised anonymity and confidentiality and we do not believe it is possible to provide transcripts of the interviews in a way that provide sufficient information to analyze the data and also maintain anonymity. As stated in the PLOS ONE policy we have excerpts of the transcripts in the paper in the form of quotes. |
| 6. Your ethics statement should only appear in the Methods section of your manuscript. If your ethics statement is written in any section besides the Methods, please delete it from any other section. | The ethics statement was supposed to be a subsection under the method section, but the level of headlines was incorrect. This has been corrected. |
| Please review your reference list to ensure that it is complete and correct. If you have cited papers that have been retracted, please include the rationale for doing so in the manuscript text, or remove these references and replace them with relevant current references. Any changes to the reference list should be mentioned in the rebuttal letter that accompanies your revised manuscript. If you need to cite a retracted article, indicate the article’s retracted status in the References list and also include a citation and full reference for the retraction notice. | The reference list has been reviewed and all webpages has been accessed again 6. November |
| **Reviewer #1:** |  |
| The submitted paper is impressively written highlighting the importance of subject matter. Methodology chapter well defined the criteria and pathway of research. Results are well explained accordance with the findings. Discussion was thoroughly written and explained with latest references. | Thank you for the nice comments. |
| **Reviewer #2: Comment 1.** | That you for the comments |
| 1. How you check the reliability of your interview guide? ( You didn't mention it) | We have elaborated on this issue in the Design and data collection section line 119-121 where we described that the interview guides were semi-structured and though fairly consistent they were adjusted a few times during the first interviews to elaborate on perspectives uncovered in the initial interviews. |
| 2. Which sampling method is employed in this study? | We have added in line 107 to the description of the sampling that is a convenience sampling. |
| 3. How you concluded that 13 GP's are sufficient for this study? | We have rephrased and clarified the description hereon. |
| 4. Were the subjects ever informed of the true nature of the study. If so when and if not then why not? | The interviewees were informed about the purpose of the study at recruitment and again in the beginning of the each interview. This information is added to the method section line 124-125. |
| Comment 2. While the study appears to be sound, the language is unclear, making it difficult to follow.  I advise the authors work with a copyeditor to improve the sentence flow and readability of the paper. | The manuscript has been in language revision and changes have been made correspondingly. |
