# Supplementary material for: Making sense of and working with COVID-19 related guidelines and information in Danish general practice–A qualitative study
Source: PLoS One. 2023 Feb 10;18(2):e0281579. doi: 10.1371/journal.pone.0281579 (PMC9916589; doi:10.1371/journal.pone.0281579)
Supplement: Attachment — Submitted filename: Response to reviewers.docx [file pone.0281579.s002.docx]

**Response to reviewers**

Our point by point responses are written below.

Thank you.

**Additional Editor Comments:**

Thank you for submitting this interesting manuscript. If you can do minor edits in English that would be even better.

**Response**: We have read the manuscript thoroughly and made relevant edits in English including those suggested by reviewer #3.

**Reviewers' comments:**

**Reviewer #2:** First of all, the overall content is not presented in an intelligible way. Secondly, there are many errors in the sentence flow and standard english. Same error as earlier. Moreover, this manuscript findings are not clear and satisfactory. So, i am not satisfied with this manuscript as this is failed in fulfilling the requirements of PLOS. Thankyou.

**Response:** We have worked through the manuscript again and made a few corrections to the language. The manuscript has been in language revision, and Reviewer #3 who is a GP from England has only minor comments to the language, so we believe the manuscript conforms to standard English.

With regard to the critique of the content and the findings, we find it difficult to respond to such general comments that contain no examples of what the reviewer considers wrong with the text. In light of the very positive comments from Reviewer #3, and the positive comments given by reviewer #1 in the first review, we have chosen not to make significant chances to the main text, as that might jeopardize their perception of the manuscript.

**Reviewer #3:** Thank you for inviting me to review this paper on 'Making sense of and working with COVID-19 related guidelines and information in Danish general practice – a qualitative study'.

I really enjoyed reading it as it resonated with my own experience as a GP in England during the first few months of the pandemic.

The methods are transparent, the findings clear and the discussion interesting which includes drawing on existing evidence from implementation science.

**Response:** Thank you for these nice comments.

**Comment:** It may be nice to have a summary box with learning points for the reader and/or perhaps recommendations for policymakers in future.

**Response:** We have made a summary box with recommendations for policy makers. We have included this in a separate file, as we will leave it to the editor to judge whether such a box is suitable in PLOS one articles.

**Comment:** As a reader from another country it would be really helpful to get a feel for the characteristics of the practice including how many patients they had and what staff worked in each practice (approx number and roles) - so that when reference is made to the practice meeting the reader can visualise who the GPs are meeting with.

**Response:** Thank you for this comment. We agree that a little more information would be useful here. We have inserted a table (table 1) with GP characteristics. We are not able to describe the staff or exact number of patients in each practice, but in we have added descriptions of the average number of patients per GP in Denmark and of the variety of Danish general practices.

**Comment:** A table with the demographics of the participants could be helpful for the reader.

**Response:** In the new table 1 mentioned above, we have included age, gender and years working as a GP for each participant. We have chosen to list age and years working as a GP as intervals to maintain anonymity.

**Comment:** The reader comes to understand that there was potentially a point in time that GPs would not be paid for a video consultation, but they eventually were - for those unfamiliar with the Danish system a couple of lines about how Danish GPs are paid would be very helpful to understand how this was even in question at some point.

**Response:** We have added some information on how the GPs are paid in general in the introduction and a sentence on remuneration for video consultations in the Setting section.

**Comment:** I was surprised to see that ethical approval was not needed as this was a 'qualitative' study. Qualitative studies in the UK would be expected to have ethics approval. Again for the non-Danish reader a sentence explaining the logic to this would be helpful.

**Response:** We are aware that the Danish procedure on ethical approval of qualitative research projects is very different from those in other countries. We do not know the logic behind the Danish procedure, but the Danish Ethics Committees state on their homepage that qualitative research and surveys do not need their approval. Recognizing that many foreign reviewers will wonder about this, it is customary for qualitative researchers in Denmark to send their research protocols to the committee to obtain a confirmation that their project does not need their approval. This was also what we did. The regulations around ethical approval of research can be read here, but unfortunately only in Danish. <https://nationaltcenterforetik.dk/ansoegerguide/overblik/hvad-skal-jeg-anmelde>

**Comment**
I noted a typo in the translation of a quote:
Pg 13, line 242 What, related to who should be send (should be sent) where?”

**Response:** Thank you for alerting us to this, we have corrected the mistake.

**Comment**
Pg 15 line 281. The use of the word hereon is unusual. I can see it has also been used elsewhere, and I would generally suggest removing it, as a native speaker it is not commonly used.

**Response:** We have followed the suggestion.

**Comment:** Well done for capturing these views during a challenging time. It was fascinating to read how much in common GPs in Denmark had with those in England.

**Response:** Thank you!
